# Electroconvulsive Therapy for Trauma-Related Nightmares: A Case Report and Commentary

**DOI:** 10.7759/cureus.16546

**Published:** 2021-07-21

**Authors:** Oliver Shore, Pauline Chen, Tessy Korah

**Affiliations:** 1 Psychiatry, University of Florida, Gainesville, USA

**Keywords:** trauma-related nightmares, post-traumatic stress disorder, electroconvulsive therapy, traumatic brain injury, trauma-focused psychological therapy

## Abstract

Trauma-related nightmares (TRN), one of the most reported symptoms of posttraumatic stress disorder (PTSD), may not always respond to current pharmacologic and therapeutic treatments. Validity of electroconvulsive therapy (ECT), which is used worldwide in clinical treatment for a broad range of neuropsychiatric conditions, is investigated as a potential therapeutic option for TRN in this report. A case of a 39-year-old male with a history of severe combat-related PTSD, major depressive disorder, history of traumatic brain injury, suicidal ideations, and persistent TRN is discussed here. Successful treatment outcome of this case with six sessions of right unilateral ECT is presented. On initial presentation, the patient had a Patient Health Questionnaire-9 (PHQ-9) score of 27 and a Posttraumatic Stress Disorder Checklist for DSM-5 (PCL-5) score of 77. After six sessions of ECT, the patient had a PHQ-9 score of 3 and a PCL-5 score of 45. Furthermore, the rationale and potential mechanisms of action underlying the ECT treatment for treatment-resistant PTSD and TRN are also reviewed in this report.

## Introduction

Trauma is an emotional response to a shocking or overwhelming event that threatens one’s physical, mental, or emotion safety. The event can be experienced first-hand, witnessed in person, happen to a close relation, or occur following repeated exposure to disturbing details of traumatic events [1]. A majority of those exposed to a traumatic event will have a psychological reaction that can manifest into four broad symptoms groups: intrusion, avoidance, hyperarousal, and negative cognition and mood. Intrusive symptoms consist of recurrent involuntary trauma-related memories, flashbacks, and nightmares that cause physiologic and psychologic distress. Avoidant symptoms are behavioral changes that are enacted to avoid internal and external trauma reminders. Hyperarousal symptoms consist of irritable and angry outbursts without appropriate provocation, reckless behavior, hypervigilance, exaggerated startle, and sleep disturbance. Negative cognition and mood symptoms can include amnesia for the traumatic event, negative emotional states, negative beliefs about oneself, others, or the world, anhedonia, and a loss of positive emotions [1]. Additionally, approximately half of people with posttraumatic stress disorder (PTSD) also have a diagnosis of major depressive disorder (MDD) [2].

Trauma-related nightmares

Trauma-related nightmares (TRN), one of the most reported symptoms of PTSD, are repeated, dysphoric dreams that cause significant distress and functional impairment [3]. Nightmares occur in association with multiple psychiatric diseases including acute stress disorder, MDD, bipolar disorder, and psychotic disorders and can be significant causes of distress [1,4]. TRN can also coincide with other sleep disturbances including insomnia due to a fear of falling asleep [5]. TRN are commonly experienced at similar rates throughout populations. Combat veterans with PTSD (52.4%) and survivors of the Oklahoma City Bombing (50%) experienced nightmares at similar rates [5,6]. Moreover, Krakow et al. found that 60% of PTSD patients reported the frequency of nightmares as many as six nights per week [7].

Physiologic mechanisms associated with nightmares and PTSD are potentially associated with limbic, paralimbic, and prefrontal dysfunction [8]. Limbic system dysfunction may lead to overactive arousal, and prefrontal dysfunction hinders appropriate de-escalation. The hippocampus and amygdala detect, generate, and maintain fearful emotions. The hippocampus maintains memories of conditioned fear, and the amygdala encodes, retrieves, and elicits a physiologic fear response [8,9]. The hippocampus and amygdala also contribute to dream production and dream content and consequently, the hippocampal dysfunction has been reported to cause short, stereotyped, and repetitive dreams. Recurrent trauma-related nightmares may be due to a dysfunctional hippocampus causing repetitive traumatic dreams and an amygdala that is sensitive and responsive to the fear-inducing elements portrayed in the dream [9].

Treatment strategies for TRN

Although treatment guidelines for PTSD vary according to each psychiatric organization, there is a general consensus that trauma-focused psychological therapy (TFPT) is effective and should be utilized as a first-line therapy. TFPT encompasses multiple therapies including exposure therapy, cognitive processing therapy, trauma-focused cognitive behavioral therapy, and eye movement desensitization and reprocessing [10].

Behavioral therapies have been found to be effective in treating TRN. Image rehearsal therapy, where participants practice envisioning pleasant imagery and use these images to modify nightmares, significantly reduced the frequency of nightmares in sexual assault survivors by 60%. However, it did not result in complete TRN remission, with patients still experiencing nightmares 1.3 nights per week [11]. Exposure, relaxation, and rescripting treatment (ERRT) is a similar behavioral therapy that focuses on modifying physiological, behavioral, and cognitive channels of anxiety before bedtime, which has been shown to be helpful in reducing TRN in several case series [12]. Selective serotonin reuptake inhibitors (SSRIs) and selective serotonin-norepinephrine reuptake inhibitors (SNRIs) have the greatest evidence supporting their use in PTSD treatment, and prazosin can be used with SSRIs or SNRIs to reduce nightmares and sleep disturbances [3]. Prazosin has been shown to decrease recurrent and distressing TRN by 50% in combat veterans with PTSD although nightmares were still experienced 2.3 nights per week [13]. Prazosin has alpha-1 adrenergic-blocking properties that antagonizes norepinephrine outflow from the amygdala and may otherwise contribute to autonomic stimulation, disrupted sleep, and corticotropin-releasing factor release [3]. Pharmacotherapy is typically used for TRN in conjunction with TFPT or when TFPT is unavailable [10]. Pharmacotherapy is generally effective at decreasing hyperarousal and mood symptoms, but is less effective at reducing intrusive and avoidant symptoms [3].

Existing pharmacologic and behavior therapies for TRN, while effective in reducing TRN frequency, rarely allow patients to achieve full remission. Patients continue to experience nightmares multiple nights a week that disrupt healthy sleep and negatively affect quality of life. Full remission of TRN remains a challenging but an important treatment goal.

Rationale for electroconvulsive therapy treatment for TRN

Electroconvulsive therapy (ECT) is a safe and effective treatment modality for a variety of psychiatric conditions including depression, mania, schizophrenia/ schizoaffective disorder, and catatonia. Diagnostic indications for ECT usage vary geographically, with schizophrenia and schizoaffective disorders being the number one indication worldwide [14].

In the United States, affective disorders are the main diagnostic indication for ECT, accounting for 72%-92% of treatments while schizophrenia and/or schizoaffective disorders account for the remainder of ECT treatments, roughly 8%-29%. Non-mood and psychotic disorders account for less than 1% of ECT treatments [14].

Patients with major depressive episodes achieve a 60% remission rate and a 70% response rate with ECT. Mania that is not responsive to pharmacologic therapy has been effectively treated with ECT and is preferred in severe mania variants like catatonic excitement [15]. Although antipsychotic medications outperform ECT in treating schizophrenia and other psychotic disorders, the combination of ECT and antipsychotic therapy appears to reduce symptom duration and improve remission rates in medication-resistant patients [3,15]. Unlike with depression and psychotic disorders, limited research has been conducted evaluating ECT for PTSD.

## Case presentation

A 39-year-old male with an approximate 13-year history of PTSD, MDD, alcohol use disorder in remission, obstructive sleep apnea (OSA), and a history of traumatic brain injury (TBI) presented for worsening depression, severe combat-related PTSD, and suicidal ideations (SI). The patient had been tried on several medications prior to admission including prazosin, doxepin, amitriptyline, bupropion, duloxetine, fluoxetine, mirtazapine, paroxetine, propranolol, quetiapine, risperidone, and topiramate without relief from his symptoms. Patient trialed prazosin for a minimum of four months and up to 25 mg without benefit. He was also engaged in group and individual therapies that included present-centered therapy (PCT), prolonged exposure (PE), trauma-focused, and acceptance and commitment therapy (ACT) for at least two years.

On initial presentation, he reported SI daily and woke up screaming from nightmares every night, which severely impacted his quality of life. On Patient Health Questionnaire-9 (PHQ-9), he scored 27 and his Posttraumatic Stress Disorder Checklist for DSM-5 (PCL-5) score was 77, indicating severe symptoms of MDD and PTSD, respectively. His medication regimen while inpatient was adjusted to include venlafaxine 225 mg daily, aripiprazole 5 mg daily, trazodone 200 mg nightly, mirtazapine 15 mg nightly, and cyproheptadine 8 mg nightly. Cyproheptadine was later switched to clonidine 0.1 mg with minimal benefit. The patient was also seen one to two times a week for individual cognitive behavioral therapy (CBT) and ACT. He additionally regularly attended group therapy sessions while inpatient. However, his depressive and PTSD symptoms did not improve significantly. He continued to report SI daily and woke up to vivid TRN. The night staff would corroborate the nighttime awakenings with reports of hearing the patient scream "abort, abort!"

Due to the severity of his depressive symptoms and despite significant improvement with therapeutic and medication management, the patient was treated with right unilateral ECT for MDD. He underwent ECT three times a week on Monday, Wednesday, and Friday. After his third ECT session, the patient reported diminished SI and no longer reported TRN. Night staff no longer reported nighttime awakenings or screaming. Furthermore, the patient reported improved quality of sleep after ECT session 3 as he was no longer waking up at night due to TRN.

ECT was greatly beneficial for the patient's depression as evidenced by the patient's report of improving SI and a PHQ-9 score of 3 on the day of discharge. By ECT session 6, the patient no longer reported SI and continued to deny reoccurrence of TRN. The patient was discharged after ECT session 6 with a plan to continue outpatient ECT sessions. On the day of discharge, his PCL-5 score was 45, which was still suggestive of PTSD symptoms, but lower than the admission score of 77 (Table 1).

**Table 1:** Patient's admission and discharge PHQ-9 and PCL-5 scores PHQ-9, Patient Health Questionnaire-9; PCL-5, Posttraumatic Stress Disorder Checklist for DSM-5

	PHQ-9	PCL-5
Admission	27	77
Discharge	3	45

## Discussion

Despite ECT’s long-time use, the mechanism of action remains largely unclarified. Theories proposed to explain the physiological basis of ECT include changes to the (a) brain morphology, (b) brain activity, (c) neurotransmitter signaling, and (d) hypothalamic-pituitary-adrenal (HPA) normalization [16-18]. Tendolkar et al. found that bitemporal ECT increased hippocampal and amygdala volume bilaterally, but could not correlate these changes with the clinical response to treatment [16]. Dukart et al. found that right unilateral ECT increased gray matter volume (GMV) throughout in the right hemisphere, and particularly in the subgenual cortex, hippocampus, amygdala, and anterior temporal pole while decreased GMV in the right prefrontal cortex [19]. Furthermore, studies have correlated improved depression scores with deactivation of the bilateral frontal and anterior temporal cortices, deactivation of the right prefrontal cortex and left posterior frontal cortex, or deactivation in the frontopolar gyrus, superior temporal gyrus, and amygdala following ECT [17]. Other studies have shown that ECT-induced seizures acutely activate the HPA axis while decreasing cortisol response to dexamethasone following ECT treatment, suggesting that ECT may normalize function by allowing for HPA suppression by the forebrain [17,18].

Considering that ECT is a safe procedure and is associated with decreased all-cause mortality in patients, it is not only a treatment modality for depression, but may also be a reasonable treatment modality for TRN, particularly in patients who do not respond to behavioral and pharmacologic therapies [14,15].

As suggested by the PCL-5 scoring, the PTSD symptoms of the patient in the current case lessened markedly after undergoing six ECT sessions. This case does not suggest complete resolution of PTSD symptoms, but does suggest benefit for TRN as the patient no longer had nighttime awakenings or reports of TRN with continued sessions of ECT. As evidenced in this case, when pharmacological and therapeutic managements have been exhausted, ECT may be a procedural option to consider for TRN. However, this therapeutic option is scarcely explored. There have only been a handful of clinical studies and case reports suggesting beneficial effects of ECT on PTSD. In a literature search by Youssef et al., in 2017, only three retrospective studies, one prospective uncontrolled clinical trial, and five case reports were found [8].

There is also consideration that there may have been improvement in TRN secondary to improvement in the patient's depressive symptoms as nightmares are not limited to PTSD [4]. However, the patient reported cessation of TRN by ECT session 3 prior to the resolution of SI. This suggests that ECT may have a direct impact on TRN rather than an indirect impact by improving depressive symptoms.

The impact of right unilateral versus bitemporal ECT for TRN and/or PTSD is unclear. However, per previous case studies and this case report, both types have been associated with the reduction of PTSD symptoms. One retrospective case control study suggested that ECT was associated with reduction in suicidality, cardiovascular, and all-cause mortality in individuals with MDD and PTSD [20].

Like with depression, the mechanism for how ECT may reduce TRN and/or PTSD symptoms is still unclear. However, one hypothesis suggests that ECT impairs reconsolidation of reactivated, emotionally aversive memories and therefore reduces PTSD symptoms [21]. Given that TRN may be due to a dysfunctional hippocampus and overactive amygdala, the reported changes caused by ECT treatment may in fact be efficacious in treating TRN [8,9,16,18]. Further research studies are needed to determine the efficacy of ECT in TRN and PTSD, as well as research in identifying the mechanisms involved.

## Conclusions

Although ECT has primarily been utilized for depression, mania, and psychotic disorders, there may be benefit in utilizing ECT for TRN. Nevertheless, ECT is generally underutilized and is often relegated to being a last-resort intervention in cases of medication non-response. However, the subjective report as well as the objective improvement per the PCL-5 scale in this case suggests that ECT can aid in the treatment of TRN. Furthermore, the impact that ECT may have on the limbic symptom suggests the potential benefit in addressing TRN that are a result of a dysfunctional limbic symptom. However, further research is needed to determine the efficacy of ECT in TRN and in determining the specific mechanisms involved in reducing nightmares.
